# PTP1B inhibitor alleviates deleterious microglial activation and neuronal injury after ischemic stroke by modulating the ER stress-autophagy axis via PERK signaling in microglia

**DOI:** 10.18632/aging.202272

**Published:** 2021-01-20

**Authors:** Yu Zhu, Jianbo Yu, Jiangbiao Gong, Jie Shen, Di Ye, Dexin Cheng, Zhikai Xie, Jianping Zeng, Kangli Xu, Jian Shen, Hengjun Zhou, Yuxiang Weng, Jianwei Pan, Renya Zhan

**Affiliations:** 1 Department of Neurosurgery, The First Affiliated Hospital, Zhejiang University School of Medicine, Hangzhou 310003, Zhejiang Province, China; 2 Emergency Department Trauma Center, The First Affiliated Hospital, Zhejiang University School of Medicine, Hangzhou 310003, Zhejiang Province, China

**Keywords:** PTP1B, endoplasmic reticulum stress, autophagy, microglia, ischemic stroke

## Abstract

Cerebral ischemia/reperfusion (IR) after ischemic stroke causes deleterious microglial activation. Protein tyrosine phosphatase 1B (PTP1B) exacerbates neuroinflammation, yet the effect of the inhibition on microglial activation and cerebral IR injury is unknown. A cerebral IR rat model was induced by middle cerebral artery occlusion (MCAO) and reperfusion. The PTP1B inhibitor, sc-222227, was administered intracerebroventricularly. Neurologic deficits, infarct volume, and brain water content were examined. An in vitro oxygen glucose deprivation/reoxygenation (OGD/R) model was established in primary microglia and BV-2 cells. Microglial activation/polarization, endoplasmic reticulum (ER) stress, autophagy, and apoptosis were detected using western blot, immunohistology, ELISA, and real-time PCR. Protein interaction was assessed by a proximity ligation assay. The results showed a significant increase in microglial PTP1B expression after IR injury. Sc-222227 attenuated IR-induced microglial activation, ER stress, and autophagy and promoted M2 polarization. Upon OGD/R, sc-222227 mitigated microglial activation by inhibiting ER stress-dependent autophagy, the effect of which was abolished by PERK activation, and PERK inhibition attenuated microglial activation. The PTP1B-phosphorylated PERK protein interaction was significantly increased after OGD/R, but decreased upon sc-222227 treatment. Finally, sc-222227 mitigated neuronal damage and neurologic deficits after IR injury. Treatment targeting microglial PTP1B might be a potential therapeutic strategy for ischemic stroke treatment.

## INTRODUCTION

Ischemic stroke is a neurologic disorder causing severe mortality and morbidity worldwide [1, 2]. While restoring cerebral blood supply by vessel recanalization is the current treatment for ischemic stroke, the reperfusion process can induce inflammation and is a major cause of neuronal injury, and it carries an unfavorable prognosis [3, 4]. Microglia, as the major resident immune cells of the central nervous system, have an important role in mediating inflammatory responses and brain injury following diverse insults. After ischemic stroke, microglia become activated and release multiple pro-inflammatory cytokines, which further result in deleterious and neurotoxic consequences [5–7]. While reducing microglial activation and inhibiting neuroinflammatory responses is considered a promising therapeutic strategy for ischemic stroke, the mechanisms underlying microglial activation after ischemic stroke are far from clear.

Protein tyrosine phosphatase 1B (PTP1B) is a member of the protein tyrosine phosphatase family and has recently attracted attention as a regulator of a variety of processes within the central nervous system. In addition to early findings of the role of PTP1B in mediating insulin signaling to regulate energy expenditure and adiposity [8, 9], recent studies further revealed that PTP1B is highly expressed in microglia [10] and is a positive regulator of neuroinflammation [11]; however, the roles of PTP1B in both ischemic stroke and microglia are unclear, and based on current evidence it is possible that inhibition of PTP1B after ischemic stroke may exert neuroprotective effects by attenuating neuroinflammation.

Endoplasmic reticulum (ER) stress has been shown to be involved in the neuronal injury after ischemia/reperfusion (IR) injury, and inhibition of ER stress effectively protects neuronal injury after ischemic stroke [12–14]. Several studies have also demonstrated the role of PTP1B in positively regulating ER stress, and inhibition of PTP1B significantly alleviated ER stress-induced neurotoxicity [15, 16]. Moreover, recent research has revealed that ER stress is involved in the microglial activation process, which causes neuroinflammation [17, 18].

Autophagy is a lysosome-mediated self-degradation process that eliminates damaged or aged proteins and organelles, and is recognized as an important element of innate immune responses [12]. Newly emerging evidence has shown that the complicated roles of autophagy are both neuroprotective or destructive in neuronal cell death [19], and various factors have been shown to be involved in modulating autophagy. Studies have revealed the role of ER stress as an upstream trigger for autophagy induction [20–23], and recent studies have further shown that the ER stress-autophagy axis is involved in acute neuronal injury caused by ischemic stroke-induced neuroinflammation [24]. In particular, the microglial ER stress-autophagy axis was shown to have a critical role in regulating microglial activation and subsequent neuroinflammation in response to external cocaine stimulation [18]. The exact role of PTP1B in ER stress and autophagy remain elusive, although a recent study showed protective effects of PTP1B deletion for myocardial injury by obliterating ER stress through regulation of autophagy [25], indicating potential involvement of PTP1B in the ER stress-autophagy axis.

Based on these findings, we determined whether pharmacologic inhibition of PTP1B might be able to suppress the microglial ER stress-autophagy axis, and we assessed the effect of PTP1B inhibition against cerebral ischemia /reperfusion (IR) injury.

## RESULTS

### Upregulation of PTP1B in microglia after rat ischemia/reperfusion injury

**Figure 1:**
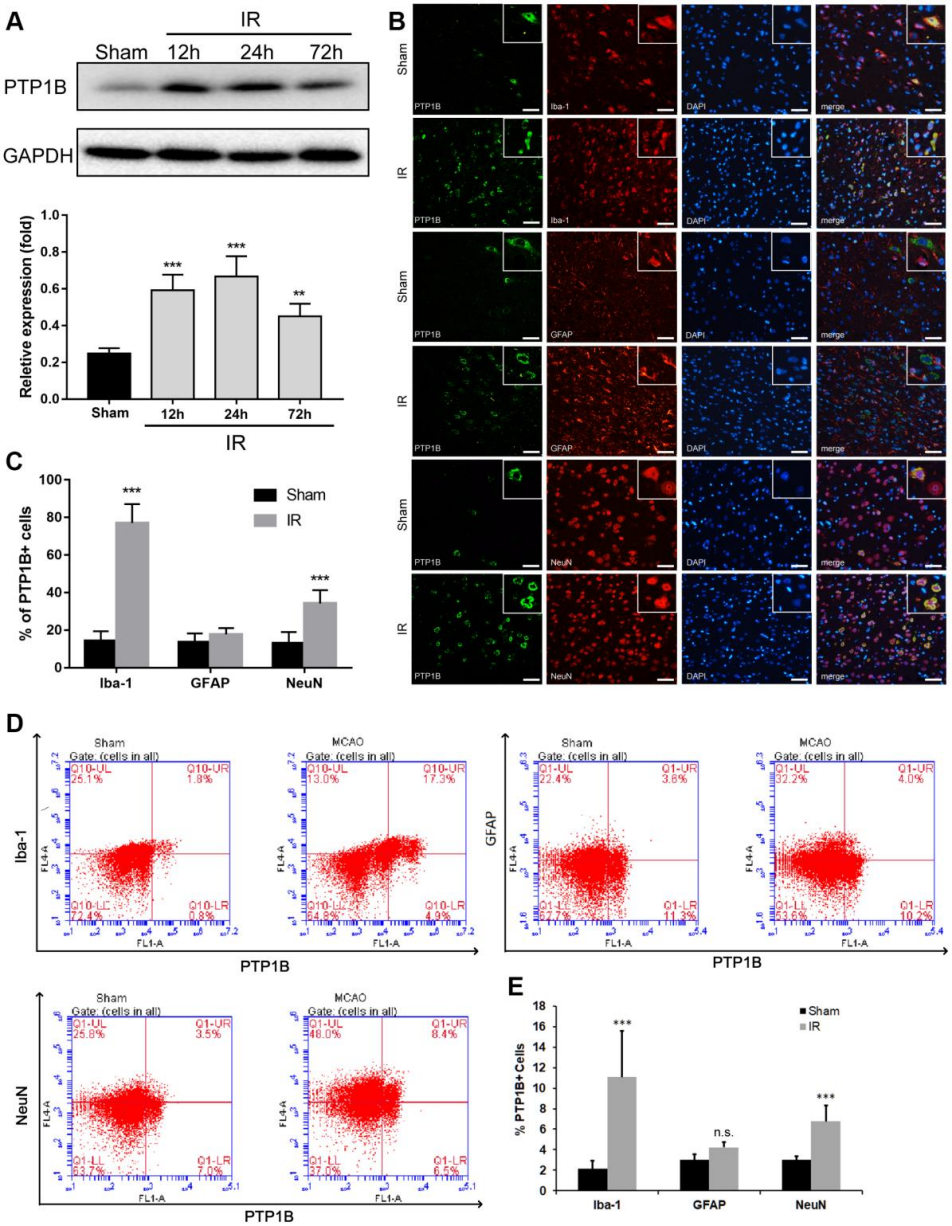
**Upregulation of PTP1B protein expression after cerebral ischemia/reperfusion (IR) injury.** (**A**) PTP1B protein was detected by western blot in the rat ipsilateral cortex 12, 24, and 72 h after cerebral IR injury, and was normalized to GAPDH. Quantitative results of relative band density are presented as the mean ± SEM (n = 4 per group). (**B**) Double immunofluorescence staining to detect cell type distribution of PTP1B in microglia (Iba-1), astrocytes (GFAP), and neurons (NeuN) in ipsilateral cerebral cortex 24 h after cerebral IR injury. Scale bar = 50 μm. (**C**) Quantitative analysis of the percentage of PTP1B-positive cell in microglia, astrocytes, and neurons after cerebral IR injury are presented as the mean ± SEM (n = 6 per group). (**D**) Flow cytometry to determine the cell type ratio of PTP1B in ipsilateral cortical microglia (Iba-1), astrocytes (GFAP), and neurons (NeuN) 24 h after cerebral IR injury. (**E**) Quantitative analysis of (**D**). The results are presented as the mean ± SEM (n = 5 per group). *p < 0.05; **p < 0.01; ***p < 0.001 compared with the sham group; IR = ischemia/reperfusion.

To determine whether the level of PTP1B expression was altered in response to cerebral IR injury, a rat model of transient middle cerebral artery occlusion (MCAO)/reperfusion was made, and western blot assays were performed. Whereas the level of PTP1B protein in the rat ipsilateral cerebral cortex was relatively low in the sham-operated control group, a significant increase occurred at 12 h, reached a maximum at 24 h, and declined 72 h after IR injury (12 and 24 h groups, P < 0.001 vs. sham group; 72 h group, P < 0.01 vs. sham group; Figure 1A). These results indicate that PTP1B expression is increased in the acute phase after IR injury.

To investigate the cell distribution of PTP1B expression after IR injury, double immunofluorescence was applied using antibodies targeting Iba-1, GFAP, and NeuN to identify microglia, astrocytes, and neurons, respectively. The ratio of Iba-1(+)/PTP1B(+), GFAP(+)/PTP1B(+), and NeuN(+)/PTP1B(+) double-positive cells was calculated based on random fields under a laser confocal microscope. PTP1B was expressed in microglia, neurons, and astrocytes in the sham cerebral cortex (Figure 1B). Twenty-four hours after cerebral IR injury, the number of PTP1B-positive cells was significantly increased in microglia and neurons, but not astrocytes, and this augmentation in the percentage of PTP1B-positive cells was more dramatic in microglia compared with neurons (Figure 1C).

To further validate the expression of PTP1B in various types of cells after cerebral IR injury, flow cytometry was used to detect the proportion of Iba-1(+)/PTP1B(+), GFAP(+)/PTP1B(+), and NeuN(+)/PTP1B(+) double-positive cells (Figure 1D). The proportion of Iba-1(+)/ PTP1B(+) and NeuN(+)/PTP1B(+) double-positive cells increased significantly after IR injury, and the proportion of Iba-1(+)/ PTP1B(+) in the IR group increased by approximately five-fold compared with the sham group (P < 0.001; Figure 1E). These findings implied that upregulation of PTP1B expression after rat IR injury was prominent in microglia. Because microglia are the main immune cells in the brain that mediate inflammatory responses after cerebral IR injury, and considering the critical role of PTP1B in regulating neuroinflammation, the above results suggested that PTP1B might contribute to the pathophysiologic process by regulating microglia function following cerebral IR injury.

### PTP1B inhibitor reduced ischemia/reperfusion-induced microglial activation and promoted M2 microglial polarization

To investigate the role of PTP1B in regulating microglial activation and polarization, we first explored the effect of a selective PTP1B inhibitor, sc-222227 (referred to as PTP1Bsc hereafter), on rat primary microglial cell activation after oxygen glucose deprivation/reoxygenation (OGD/R) injury. Primary microglial cells were maintained in glucose-free medium in an anaerobic chamber for 3 h and returned to normal cell culture incubators with normal medium. The PTP1B inhibitor, sc-22227, was added to microglial cultures 2 h before OGD/R treatment.

**Figure 2:**
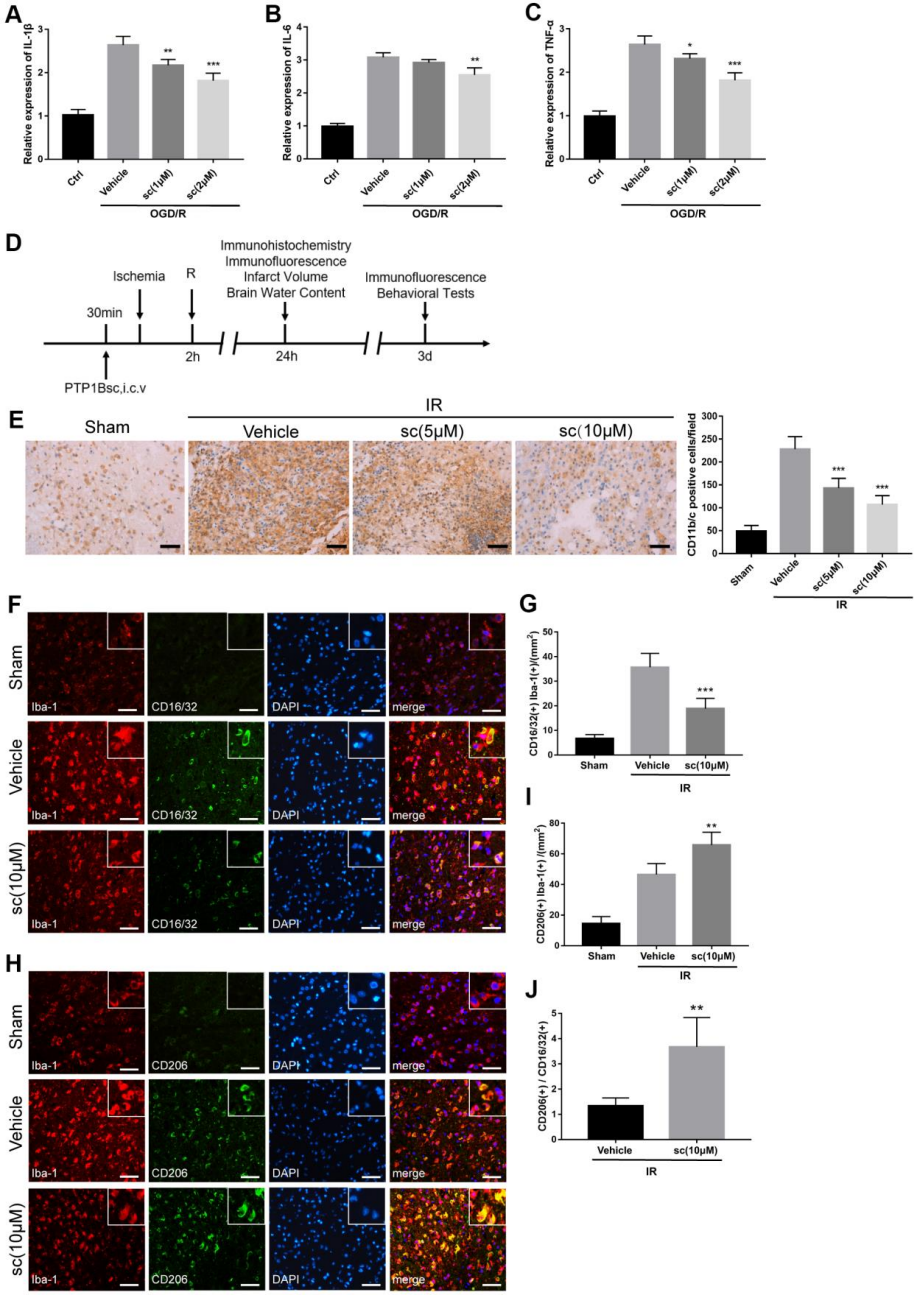
**PTP1B inhibitor treatment attenuated microglial activation and promoted M2 microglial polarization after ischemic injury.** (**A**–**C**) IL-1β, IL-6, and TNF-α mRNA levels after the OGD/R insult were tested by real-time PCR in rat primary microglia. Fold-changes were normalized to β-actin and quantitative results are presented as the mean ± SEM (n = 5 per group). (**D**) Outline of *in vivo* experiment to detect the effect of intracerebroventricular administration of PTP1B inhibitor after cerebral IR injury. (**E**) Immunohistology to detect CD11b/c cell in the ipsilateral cerebral cortex, and quantitative analysis of CD11b/c-positive cell number are presented as the mean ± SEM (n = 5 per group). Scale bar = 50 μm. (**F**, **G**) Double immunofluorescence to detect CD16/32(+) Iba-1(+) cell in ipsilateral cerebral cortex 72 h after IR injury, and quantitative analysis of CD16/32(+) Iba-1(+) cell density (presented as the mean ± SEM, n = 6 per group). Scale bar = 50 μm. (**H**, **I**) Double immunofluorescence to detect CD206(+) Iba-1(+) cells in the ipsilateral cerebral cortex 72 h after IR injury, and quantitative analysis of CD206(+) Iba-1(+) cell density (presented as the mean ± SEM, n = 6 per group). Scale bar = 50 μm. (**J**) Quantitative analysis of the ratio of Iba1(+)/CD206(+) microglia to Iba-1(+)/CD16/32(+) microglia; the results are presented as the mean ± SEM. *p < 0.05; **p < 0.01; ***p < 0.001 compared with vehicle group; sc = sc-222227, a PTP1B inhibitor; i.c.v. = intracerebroventricular injection; IR = ischemia/reperfusion; R = reperfusion; OGD/R = oxygen glucose deprivation/reoxygenation.

We found that 2 μM of PTP1Bsc treatment significantly decreased the mRNA levels of IL-1β, IL-6, and TNF-α (relative mRNA expression level: IL-1β, P < 0.001; IL-6, P < 0.01; TNF-α, P < 0.001; all compared to vehicle groups; Figure 2A–2C) in rat primary microglial cells 24 h after OGD/R insult. An *in vivo* experiment (outlined in Figure 2D) further showed that intraventricular administration of both 5 and 10 μM PTP1B inhibitor 30 min prior to IR injury effectively decreased the number of activated microglia (CD11b/c+) in the ipsilateral cerebral cortex after cerebral IR injury (P < 0.001 vs. vehicle group, Figure 2D, 2E). With prolonged reperfusion (72 h, Figure 2D), 10 μM PTP1Bsc treatment significantly reduced the number of Iba1(+) CD16/32(+) cells (M1 microglia; P < 0.001 vs. vehicle group, Figure 2F, 2G), while the number of Iba1(+)/CD206(+) cells was significantly increased (M2 microglia; P < 0.01 vs. vehicle group, Figure 2H, 2I) as well as the M2-to-M1 ratio (P < 0.01 vs. vehicle group, Figure 2J) in the ipsilateral cerebral cortex.

### PTP1B inhibitor attenuated cerebral ischemia/reperfusion-induced overall and microglial endoplasmic reticulum stress in rats

**Figure 3:**
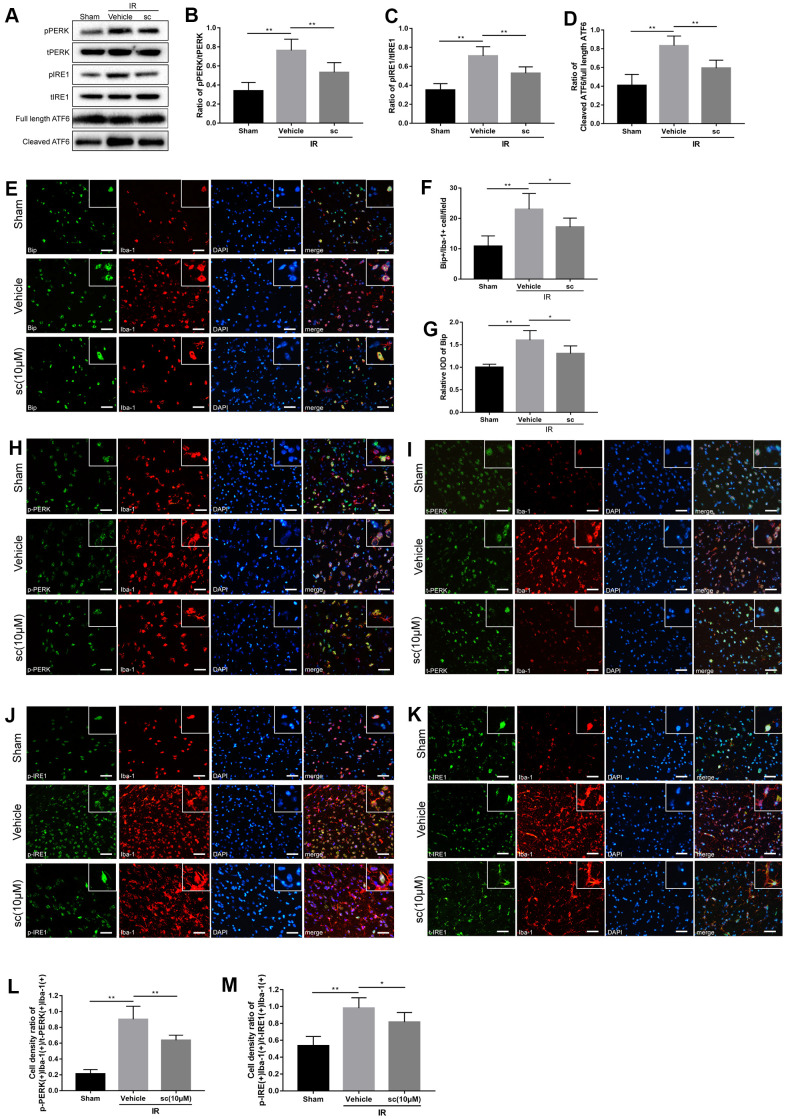
**Microglial endoplasmic reticulum (ER) stress was mitigated by PTP1B inhibitor treatment after cerebral ischemia/reperfusion (IR) injury in rats.** (**A**) Western blot to detect the effect of PTP1B inhibitor (10 μM) on phospho-PERK, total-PERK, phospho-IRE1, total-IRE1, cleaved ATF6, and full length ATF6 protein expression in the rat ipsilateral cortex 24 h after IR injury (n = 5 per group). (**B**–**D**) Quantitative analysis of band density ratio of phosphor-PERK/total-PERK, phospho-IRE1/total-IRE1, and cleaved ATF6/full length ATF6. Results are presented as the mean ± SEM (n = 4 per group). (**E**) Double immunofluorescence to detect Bip expression in microglia (Iba-1) 24 h after IR injury. Scale bar = 50 μm. (**F**, **G**) Quantitative analysis of Bip+/Iba-1+ cell density and relative integrated optical density (IOD) of Bip in the ipsilateral cerebral cortex (presented as the mean ± SEM, n = 5 per group). (**H**–**K**) Double immunofluorescence to detect phospho-PERK, PERK, phospho-IRE1, and IRE1 expression in microglia (Iba-1) 24 h after IR injury. Scale bar = 50 μm. (**L**, **M**) Quantitative analysis of double-positive cell density ratio of p-PERK(+)Iba-1(+)/PERK(+)/Iba-1(+) and p-IRE1(+)Iba-1(+)/IRE1(+)Iba-1(+) in the ipsilateral cerebral cortex (presented as the mean ± SEM, n = 6 per group). *p < 0.05; **p < 0.01; ***p < 0.001 compared with vehicle group; p-PERK = phospho-PERK; t-PERK = total PERK; p-IRE1 = phospho-IRE1; t-IRE1 = total IRE1; sc = PTP1B inhibitor sc-222227; IOD = integrated optical density; IR = ischemia/reperfusion.

Endoplasmic reticulum (ER) stress is involved in microglial activation [17, 18, 26], and PTP1B recently emerged as an important regulator that promotes ER stress in microglia [27]. ER stress activates three transmembrane stress sensors of the unfolded protein response (UPR) [PERK, IRE1, and ATF6], and while PERK and IRE1 were activated through phosphorylation, ATF6 was hydrolyzed to the cleaved form for activation [28]. To determine the involvement of the ER stress pathway in cerebral IR injury and the role of the PTP1B inhibitor in modulating microglial ER stress after IR injury, western blot assays were performed (Figure 3A) to detect ER stress sensors (total and phosphorylated PERK and IRE1, full-length and cleaved ATF6) in the sham surgery group, cerebral IR injury group with vehicle, and IR with PTP1Bsc treatment group in rats.

IR injury induced significant activation of PERK, IRE1, and ATF6 in the ipsilateral cerebral cortex (Figure 3B–3D), indicating induction of ER stress after IR injury. PTP1Bsc (10 μM) treatment dramatically attenuated IR-induced ER stress (protein level of phospho-PERK/total-PERK, P < 0.01; protein level of phospho-IRE1/total-PERK, P < 0.01; protein level of cleaved AFT6/full-length ATF6, P < 0.01; all compared to vehicle groups; Figure 3B–3D).

To further investigate the cerebral IR-induced ER stress change in microglia, another ER stress marker, the heavy chain binding protein (Bip), was used and double immunofluorescence detected Bip(+)/Iba-1(+), phospho-PERK(+)/Iba-1(+), total-PERK(+)/Iba-1(+), phospho-IRE1(+)/Iba-1(+), and total-IRE1(+)/Iba-1(+) in the sham, vehicle, and PTP1Bsc (10 μM) treatment groups (Figure 3E, 3H–3K). Cell density was calculated by double-positive cell counting per field. Cerebral IR injury significantly enhanced the cell density of Bip(+)Iba-1(+), relative overall IOD of Bip, as well as the cell density ratio of phospho-PERK(+)Iba1-1(+)/total-PERK(+)Iba-1(+) and phospho-IRE1(+)Iba1-1(+) / total-IRE1(+)Iba-1(+) (P < 0.01, all compared to the sham group; Figure 3F, 3G, 3L, 3M) in the ipsilateral rat cerebral cortex, indicating a significant elevation of microglial and overall ER stress after IR injury. PTP1Bsc (10 μM) treatment significantly mitigated IR injury-induced microglial and overall ER stress, evidenced by decreased cell density of Bip(+)Iba-1(+) [P < 0.05 vs. vehicle group, Figure 3F], relative overall IOD of Bip [P < 0.05 vs. vehicle group, Figure 3G], and decreased cell density ratio of p-PERK(+)Iba1-1(+)/t-PERK(+)Iba-1(+) [P < 0.01 vs. vehicle group, Figure 3L] and p-IRE1(+)Iba1-1(+) /t-IRE1(+)Iba-1(+) [P < 0.05 vs. vehicle group, Figure 3M].

### PTP1B inhibitor alleviated ischemia/reperfusion-induced overall and microglial autophagy

**Figure 4:**
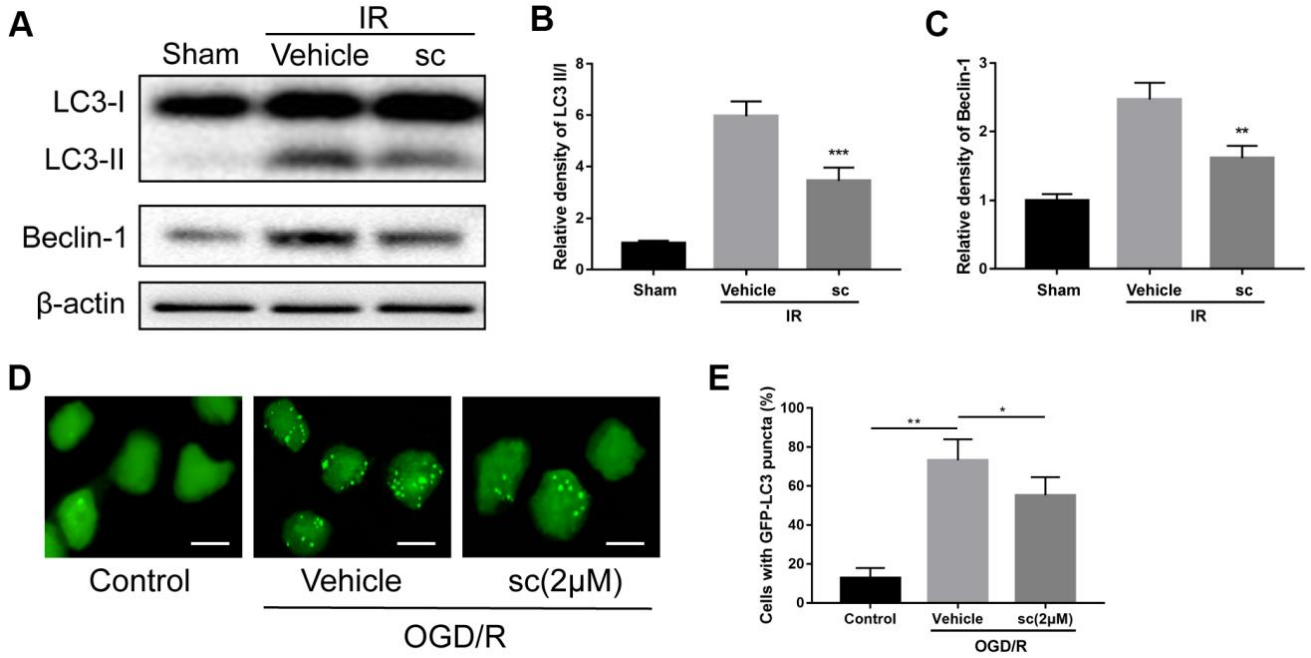
**Microglial autophagy was mitigated by PTP1B inhibitor treatment after IR injury.** (**A**) Western blot to detect the effect of PTP1B inhibitor (10 μM) on LC3-I/II and beclin-1 protein expression in the rat ipsilateral cortex 24 h after IR injury. (**B**, **C**) Quantitative results of relative band density are normalized to β-actin and presented as the mean ± SEM (n = 4 per group). (**D**) Immunofluorescence to detect GFP-LC3B puncta in GFP-LC3 expressing BV-2 cells. Scale bar = 200 μm. (**E**) Graph showing percentage of cells with GFP-LC3 puncta (presented as the mean ± SEM, n = 6 per group). *p < 0.05; **p < 0.01; ***p < 0.001 compared with vehicle group; sc = PTP1B inhibitor sc-222227; IR = ischemia/reperfusion; OGD/R = oxygen glucose deprivation/reoxygenation.

Autophagy has been demonstrated to be involved in the microglia-induced inflammatory response after ischemic stroke [29]. Reports showed that excessive autophagy contributes to neuronal death after ischemia injury [30], and suppression of autophagy in microglia effectively suppressed ischemia-induced inflammatory response [31]. To investigate the role of PTP1B inhibitor in overall autophagy after IR injury, western blot assays (Figure 4A) were performed and showed that because cerebral IR injury clearly enhanced the ratio of LC3-II/I and beclin-1 expression, treatment of PTP1Bsc (10 μM) significantly attenuated the IR-induced increase in the LC3-II/I ratio as well as beclin-1 level (LC3-II/I ratio, P < 0.001 vs. vehicle group, Figure 4B; beclin-1, P < 0.01 vs. vehicle group, Figure 4C) in the rat ipsilateral cortex.

To determine the effect of PTP1B inhibitor on ischemia-induced microglial autophagy, GFP-LC3 stable-expressing BV-2 cells were generated, and autophagy status was evaluated by calculating the percentage of cells containing GFP-LC3 puncta. Three hours after OGD/R injury, a dramatic increase in GFP-LC3 puncta was observed, and 2 μM PTP1Bsc treatment significantly reduced the accumulation of GFP-LC3 puncta (P < 0.05 vs. vehicle group; Figure 4D, 4E), indicating effective attenuation of OGD/R-induced microglial autophagy. Together, these data suggest that PTP1B inhibitor treatment significantly alleviated IR-induced overall and microglial autophagy.

### PTP1B inhibitor mitigated OGD/R-induced microglial activation through inhibition of ER stress-dependent autophagy in primary microglia with involvement of PERK signaling

**Figure 5:**
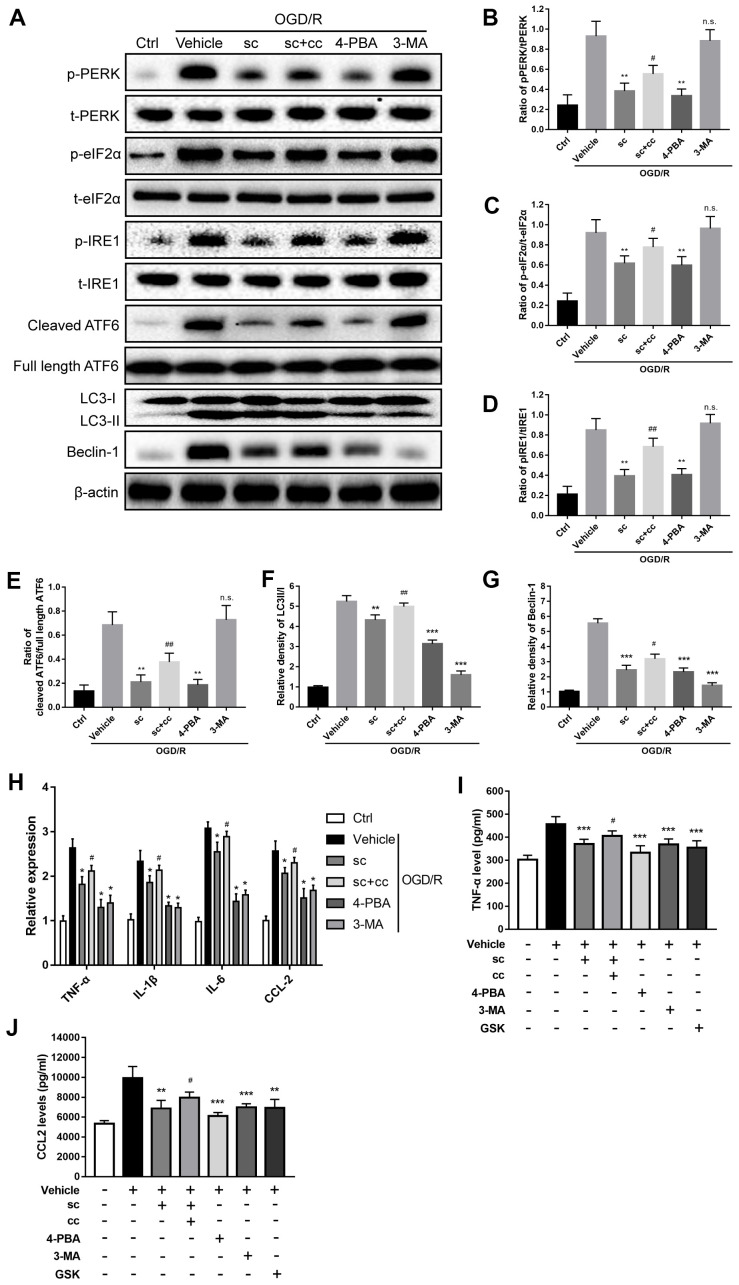
**PTP1B inhibitor mitigated oxygen glucose deprivation/reoxygenation (OGD/R)-induced microglial activation by inhibiting ER stress-dependent autophagy via PERK signaling.** (**A**) The PTP1B inhibitor, sc-222227, 4-PBA, and 3-MA were used to specifically inhibit PTP1B, ER stress, and autophagy in primary microglia, respectively. CCT020312 was used to activate PERK. GSK2606414 was used to inhibit PERK. Western blots were performed to detect p-PERK, PERK, p-eIF2α, eIF2α, p-IRE1, IRE1, cleaved ATF6, full length ATF6, LC-3I/II, and beclin-1 in primary microglia after OGD/R insult. (**B**–**G**) Quantitative results of the band density ratio of phosphor-PERK/total-PERK, phospho-eIF2α/total- eIF2α, phospho-IRE1/total-IRE1, and cleaved ATF6/full length ATF6; the relative band density of LC3II/I and beclin-1 are normalized to β-actin. The results are presented as the mean ± SEM (n = 4 per group). (**H**) Real-time PCR results showing the relative expression of TNF-α, IL-1β, IL-6, and CCL2 in primary microglia in response to treatment with PTP1B inhibitor, PTP1B inhibitor+PERK activator, 4-PBA, 3-MA, and PERK inhibitor after OGD/R insult. Data are normalized to β-actin and presented as the means ± SEM (n = 4 per group). (**I**, **J**) ELISA assay to detect expression of secreted TNF-α and CCL2 in primary microglia supernatant 24 h after OGD/R insult. Data are expressed as the means ± SEM (n = 4 per group). *p < 0.05; **p < 0.01; ***p < 0.001 compared with vehicle group; #*p* < 0.05; ##*p* < 0.01; ###*p* < 0.001 compared with the PTP1B inhibitor group; sc = PTP1B inhibitor, sc-222227; cc = PERK activator, CCT020312; GSK = PERK inhibitor, GSK2606414; p-PERK = phospho-PERK; t-PERK = total-PERK; p-eIF2α = phospho-eIF2α; t-eIF2α = total-eIF2α; p-IRE1 = phospho-IRE1; t-IRE1 = total-IRE1; OGD/R = oxygen glucose deprivation/reoxygenation.

It has been reported that generation of autophagy is triggered by upstream ER stress pathways [24, 32, 33], and the ER stress-autophagy axis is involved in cocaine-induced microglial activation [18]. First, to determine the role of ER stress in the induction of autophagy in the context of IR injury, as well as its effect on OGD/R-induced microglial activation, the specific ER stress inhibitor, 4-phenyl butyrate (4-PBA), and the specific autophagy inhibitor, 3-methyladenine (3-MA), were applied. Western blot analysis showed that OGD/R injury resulted in significant upregulation of ER stress and autophagy activity (Figure 5A, 5B, 5D–5G) in primary microglia. Suppression of ER stress by 4-PBA not only inhibited OGD/R-induced upregulation of the phospho-PERK/total-PERK, phospho-IRE1/total-IRE1, and cleaved ATF6/full length ATF6 ratios (P < 0.01, all compared to vehicle group; Figure 5B, 5D, 5E), but also suppressed OGD/R-induced upregulation of autophagy as evidenced by the decreased ratio of LC3-II/I (P < 0.001 vs. vehicle group; Figure 5F) and beclin-1 protein ratios (P < 0.001 vs. vehicle group; Figure 5G); however, 3-MA treatment only attenuated autophagy (LC3-II/I ratio: P < 0.001 vs. vehicle group, Figure 5F; beclin-1: P < 0.001 vs. vehicle group, Figure 5G), but had no significant effects on ER stress proteins (P>0.05 vs. vehicle groups for phospho-PERK/total-PERK, phospho-IRE1/total-IRE1, and cleaved ATF6/full length ATF6 ratios; Figure 5B, 5D, 5E). Then, we examined the role of the microglial ER stress-autophagy axis on microglial activation after an OGD/R insult. Both 4-PBA and 3-MA significantly attenuated IR injury-induced microglial activation, as evidenced by a significant decrease in TNF-α, IL-1β, IL-6, and CCL-2 mRNA expression (P < 0.05 vs. vehicle group; Figure 5H) as well as a decline in the levels of TNF-α (P < 0.001 vs. vehicle group; Figure 5I) and CCL2 protein (P < 0.001 vs. vehicle group; Figure 5J). Together, these results indicated that microglial ER stress is the upstream event after OGD/R insult, and the ER stress-autophagy axis mediates OGD/R-induced microglial activation.

PTP1B has been shown to be a negative modulator of ER stress as well as the ER stress-autophagy axis, and PERK signaling has been shown to be critical for triggering the ER stress-autophagy axis [18, 25, 32]. To further evaluate the effect of the PTP1B inhibitor on OGD/R-induced microglial activation, the microglial ER stress-autophagy axis and its underlying signaling, the PTP1B inhibitor (PTP1Bsc), a selective PERK activator (CCT020312), and a selective PERK inhibitor (GSK2606414) were applied. Western blot analysis (Figure 5A) showed that PTP1Bsc significantly decreased OGD/R-induced upregulation of the phospho-PERK/total-PERK, phospho-IRE1/total-IRE1, phospho-eIF2α/total- eIF2α, and cleaved ATF6/full length ATF6 ratios (P < 0.01 all compared to vehicle group; Figure 5B, 5D, 5E), autophagy level (LC3II/I ratio: P < 0.01 vs. vehicle group; Figure 5F and beclin-1: P < 0.001 vs. vehicle group; Figure 5G) as well as microglial activation (TNF-α, IL-1β, IL-6, and CCL-2 mRNA expression: P < 0.05 vs. vehicle group; Figure 5H, TNF-α protein level: P < 0.001 vs. vehicle group; Figure 5I, and CCL2 protein level, P < 0.01 vs. vehicle group; Figure 5J). PTP1Bsc also significantly attenuated OGD/R-induced phosphorylation of eIF2α (P < 0.01 vs. vehicle group; Figure 5C), which is a key downstream element in the PERK signaling; however, these effects of PTP1B inhibitor to suppress OGD/R-induced ER stress, autophagy, and PERK signaling were significantly abolished by co-administration of CCT020312 (phospho-PERK/total-PERK ratio, P < 0.05 vs. PTP1Bsc group; Figure 5B, phospho-eIF2α/total-eIF2α, P < 0.05 vs. PTP1Bsc group; Figure 5C, phospho-IRE1/total-IRE1 ratio, P < 0.01 vs. PTP1Bsc group; Figure 5D, cleaved ATF6/full length ATF6 ratio, P < 0.01 vs. PTP1Bsc group; Figure 5E, LC3II/I ratio, P < 0.01 vs. PTP1Bsc group; Figure 5F, and beclin-1, P < 0.05 vs. PTP1Bsc group; Figure 5G). Real-time PCR and ELISA further showed that inhibition of PERK signaling via GSK2606414 effectively inhibited OGD/R-induced microglial activation (TNF-α, IL-1β, IL-6, and CCL-2 mRNA expression, P < 0.05 vs. PTP1Bsc group; Figure 5H, TNF-α protein expression, P < 0.001 vs. PTP1Bsc group; Figure 5I, and CCL2 protein expression, P < 0.01 vs. PTP1Bsc group; Figure 5J), and co-administration of CCT020312 also partly abolished the PTP1Bsc effects on inhibiting OGD/R-induced microglial activation (TNF-α, IL-1β, IL-6, and CCL-2 mRNA expression, P < 0.05 vs. PTP1Bsc group; Figure 5H and TNF-α and CCL2 protein expression, P < 0.05 vs. PTP1Bsc group; Figure 5I, 5J). These results indicated that the PTP1B inhibitor effectively attenuated OGD/R-induced microglial ER stress, autophagy, and microglial activation through PERK signaling.

**Figure 6:**
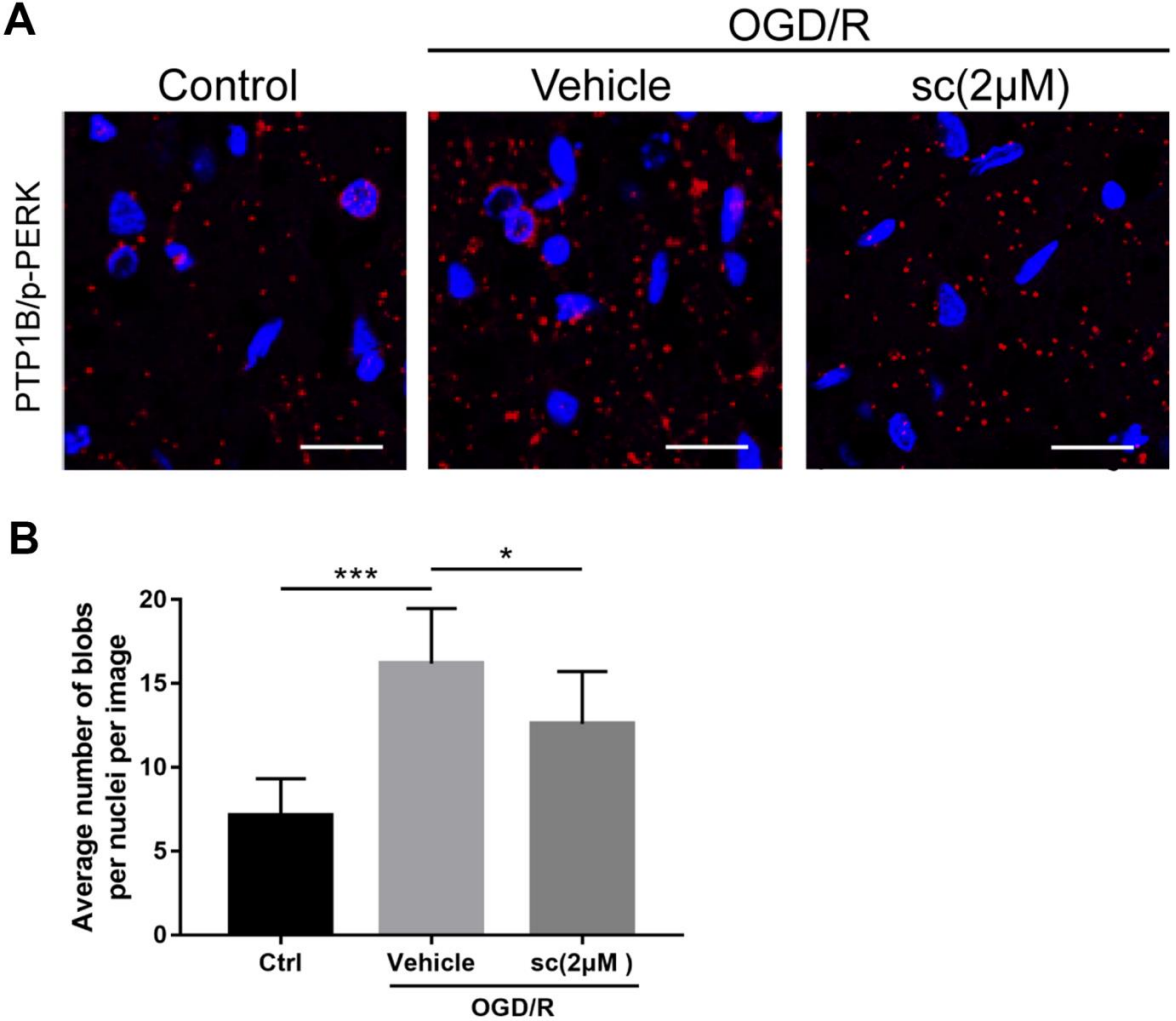
**PTP1B inhibitor treatment weakened the protein interaction between PTP1B and phospho-PERK in primary microglia under oxygen glucose deprivation/reoxygenation (OGD/R) conditions.** (**A**) Proximity ligation assay (PLA) probe was used to examine the proximity between PTP1B and phospho-PERK. Scale bar = 20 μm. (**B**) The average number of blobs per nuclei per image was quantified as the mean ± SEM (n = 6 per group). *p < 0.05 compared with vehicle group; ***p < 0.001 compared with sham group; sc = PTP1B inhibitor sc-222227; p-PERK = phospho-PERK; OGD/R = oxygen glucose deprivation/reoxygenation.

Together, the above data suggested that PTP1B inhibition by PTP1Bsc mitigated microglial activation by inhibiting endoplasmic reticulum stress-dependent autophagy in primary microglia, and PERK signaling is involved in this process.

### OGD/R injury increased the interaction between PTP1B and phosphorylated PERK in primary microglia

Because the interaction between PTP1B and phospho-PERK had been reported in several tissue types to trigger ER stress [34], we used the proximity ligation assay (PLA) probe to determine the protein interaction between PTP1B and phospho-PERK in primary microglia upon OGD/R injury, and the effect of PTP1B inhibition on the protein interaction. PLA is a technique capable of identifying interactions between two proteins in fixed tissue and cell samples. By using the corresponding primary and secondary antibodies (PLA probes) that bind to primary antibodies, the protein interaction can be detected if two proteins are in close association (when PLA probes are in close proximity [< 40 nm]) to generate a fluorescent signal and visualization as fluorescent blobs, which can be further quantified on microscopy [35].

Results showed a basal weak interaction between PTP1B and p-PERK in primary microglia (Figure 6A), whereas the interactions were markedly enhanced after an OGD/R insult, as evidenced by a significant increase in the average number of red fluorescent dots per cell per image (P < 0.001 vs. control group; Figure 6A, 6B). Interestingly, 2 μM PTP1Bsc treatment significantly weakened this protein interaction (P < 0.05 vs. vehicle group; Figure 6A, 6B). These data further support the finding that PTP1B inhibition by PTP1Bsc triggers downstream events, possibly by targeting PERK.

### PTP1B inhibitor protected against cerebral ischemia/reperfusion injury and confers neuroprotection in rats

Finally, we examined the therapeutic effects of intracerebroventricular administration of PTP1Bsc for cerebral IR-induced neuronal damage and neurologic function deficits. To achieve optimal treatment, diverse administration time points, as well as diverse doses of PTP1Bsc were tested, and intracerebroventricular injection 30 min prior to cerebral IR injury with 5 and 10 μM doses of PTP1Bsc were selected. Rats were sacrificed for immunofluorescence, infarct volume assessment (2, 3, 5-triphenyltetrazolium chloride [TTC] staining), and brain water content measurement at 24 h.

**Figure 7:**
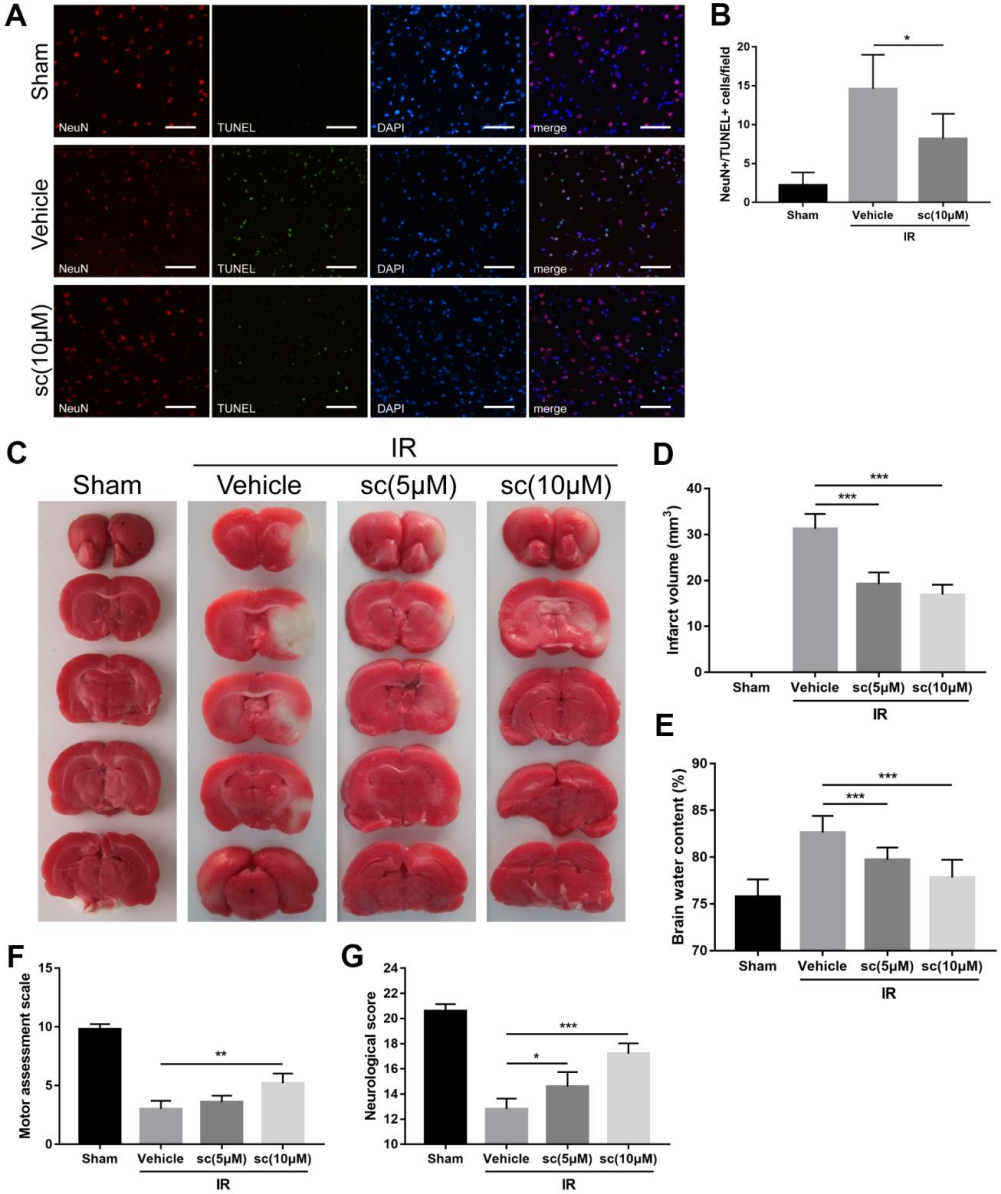
**PTP1B inhibitor treatment reduced neuronal apoptosis, cerebral infarct volume, brain water content, and improved neurologic function after ischemia/reperfusion (IR) injury.** (**A**) Immunofluorescence/TUNEL assays were performed to detect neuronal apoptosis in rat ipsilateral cerebral cortex 24 h after IR injury. Scale bar = 50 μm. (**B**) The density of NeuN+/TUNEL+ cells was quantified as the mean ± SEM (n = 6 per group). (**C**) TTC staining were performed to determine infarct volume 24 h after cerebral IR injury. Infarct area was defined as the white area. (**D**) The infarct volume was quantified as (infarct volume/whole brain volume) × 100% (n = 5 per group). (**E**) Brain water content. (**F**) Motor assessment score 3 days after IR injury. (**G**) Neurologic score 3 days after IR injury. Data for d-g are presented as the mean ± SEM (n = 6 per group). *p < 0.05; **p < 0.01; ***p < 0.001 compared with vehicle group; PTP1Bsc = PTP1B inhibitor sc-222227; IR = ischemia/reperfusion; sc = PTP1B inhibitor sc-222227.

Terminal deoxynucleotidyl transferase dUTP nick end labeling (TUNEL) assay was performed to identify cells undergoing apoptosis. PTP1Bsc significantly attenuated neuronal death 24 h after IR injury compared with the IR group (P < 0.05 vs. vehicle group, Figure 7A, 7B). Infarct volume assessment by TTC staining and measurement of brain water content were performed to evaluate overall neuronal damage. Both 5 and 10 μM of PTP1Bsc administration significantly diminished infarct volume (P < 0.001 vs. vehicle group; Figure 7C, 7D) and brain water content (P < 0.001 vs. vehicle group; Figure 7E) 24 h after IR injury. Finally, neurologic function after IR injury was assessed by behavioral tests using neurologic [24] (a 21-point Garcia test score system; the higher the score, the better the neurologic function) and motor assessment scores [36] (a 10-point score system; the higher the score, the better the motor function). PTP1Bsc administration significantly increased the motor assessment (P < 0.01, 10 μM group vs. vehicle group; Figure 7F) and neurologic scores (P < 0.05, 5 μM group vs. vehicle group and P < 0.001, 10 μM group vs. vehicle group; Figure 7G) 3 days after the IR injury. Together, these data demonstrated that PTP1Bsc treatment has a neuroprotective effect against cerebral IR injury.

## DISCUSSION

Deleterious microglial activation has been shown to cause neuroinflammation that exacerbates neuronal damage. Diverse treatments to inhibit microglial activation have been associated with improved outcome after ischemic stroke [37–39]. In this study, we demonstrated that pharmacologic inhibition of PTP1B in microglia effectively reduced detrimental microglial activation, attenuated inflammatory response and protected neuronal death after cerebral IR injury. We also provided evidence that ER stress-autophagy axis via PERK signaling is involved in the protective effect of PTP1B inhibition in microglia, indicating a novel mechanism for PTP1B in regulation of microglial activation and neuroinflammation.

PTP1B has been shown to be involved in inflammatory responses to a variety of injuries, including spinal cord and radiation injuries [40, 41]. In the current study, we first observed significant upregulation of the PTP1B level after cerebral IR injury, and the most prominent enhancement of PTP1B expression was found in microglia. Further, the time-dependent expression of PTP1B indicated an acute-phase participation of PTP1B in the acute pathophysiologic process of cerebral IR injury. Because deleterious microglial activation causing an excessive inflammatory response has also been shown to occur in the acute and sub-acute phases after IR injury [42, 43], our preliminary findings indicated a potential role for PTP1B in regulating IR-induced deleterious microglia activation.

To investigate the possible effect of PTP1B inhibition in suppression of detrimental microglial activation after cerebral IR injury, a selective PTP1B inhibitor, sc-222227, was used as a pharmacologic treatment in primary microglia OGD/R and rat cerebral IR models. The results showed that in OGD/R-treated primary microglia and IR-treated rats, inhibition of PTP1B not only effectively suppressed microglial activation and subsequent release of pro-inflammatory cytokines, but also facilitated M2 microglial polarization. These findings are consistent with previous studies that PTP1B is a positive regulator of microglial activation and polarization [11, 44–46], and our results further confirmed this crucial role of PTP1B in the context of cerebral IR injury.

Recent studies have shown that extensive involvement of ER stress in microglial activation causes cerebral injuries in Alzheimer’s disease [47], spinal cord injuries [48], and LPS/cocaine-induced neuroinflammation [17, 49], and PTP1B has been reported to promote ER stress [15, 16]. In the current study, increased PTP1B expression in the ipsilateral cortex was synchronous with the activation of UPR after IR injury [24], and intracerebroventricular administration of PTP1B inhibitor significantly attenuated IR injury-induced overall ER stress. Further, double immunofluorescence labeling of ER stress proteins and microglia showed a significant increase of ER stress in microglia after IR injury, and the IR-induced microglial ER stress was effectively attenuated by PTP1B inhibitor treatment. These findings indicated that the inhibition of PTP1B may exert its effect by mitigating ER stress in microglia.

Autophagy is a crucial cellular catabolic pathway that maintains cellular homeostasis and cell survival. The exact role of autophagy in cerebral IR injury has yet to be elucidated [50]. While several studies have shown a protective effect of autophagy against cerebral IR injury [51], many studies have reported that excessive autophagic activation exacerbates neuronal injury, and inhibition of autophagy decreases infarct size and increases the neurologic score after cerebral ischemia [24, 52]. In this study, results showed significant enhancement of autophagy proteins in the ipsilateral cerebral cortex in IR-injured rats and OGD/R-treated microglia cells, which is consistent with the results of previous studies [31, 53]. Further, IR-induced excessive autophagy in the ipsilateral cerebral cortex and microglia cells was significantly attenuated by treatment of PTP1B inhibitor, suggesting that the effect of PTP1B inhibition treatment after IR injury may suppress autophagy in microglia.

Studies have demonstrated the involvement of ER stress in the induction of autophagy, and this ER stress-autophagy axis was further shown to contribute to microglial activation. In this report, we provided evidence that after an OGD/R insult, ER stress-autophagy axis in microglia was associated with microglial activation and subsequent neuroinflammation, and inhibition of microglial PTP1B effectively suppressed microglial activation and neuroinflammation by inhibiting the ER stress-autophagy axis.

PERK signaling is an important pathway regulating ER stress, and several studies have shown a regulatory role for PTP1B in PERK-mediated ER stress [34]. In the current study, we further demonstrated that PERK signaling participates in OGD/R-induced microglial activation and neuroinflammation responses following PTP1B inhibitor treatment. In agreement with the results of previous studies, PTP1B interacts with PERK to participate in modulation of ER stress in mesenteric arteries and brown adipose tissue [34, 54]. The PLA probe assay in the current study also showed a significant enhanced interaction between PTP1B and activated PERK in primary microglia cells after an OGD/R insult, and the interaction was effectively weakened upon PTP1B inhibitor treatment. In the current study, we also observed that, with the exception of PERK signaling, other ER stress pathways (ATF6 and IRE1) were also downregulated following PTP1B inhibitor treatment, indicating complicated mechanisms are involved in the regulation of ER stress-autophagy axis by PTP1B inhibitor treatment after IR injury, which needs to be confirmed by further experiments.

Finally, we demonstrated that intracerebroventricular PTP1B inhibitor treatment alleviated neuronal apoptosis, reduced neuronal damage, and protected neurologic function after cerebral IR injury in rats. These findings for the protective effect of PTP1B inhibition were consistent with the results of a previous study [55].

**Figure 8:**
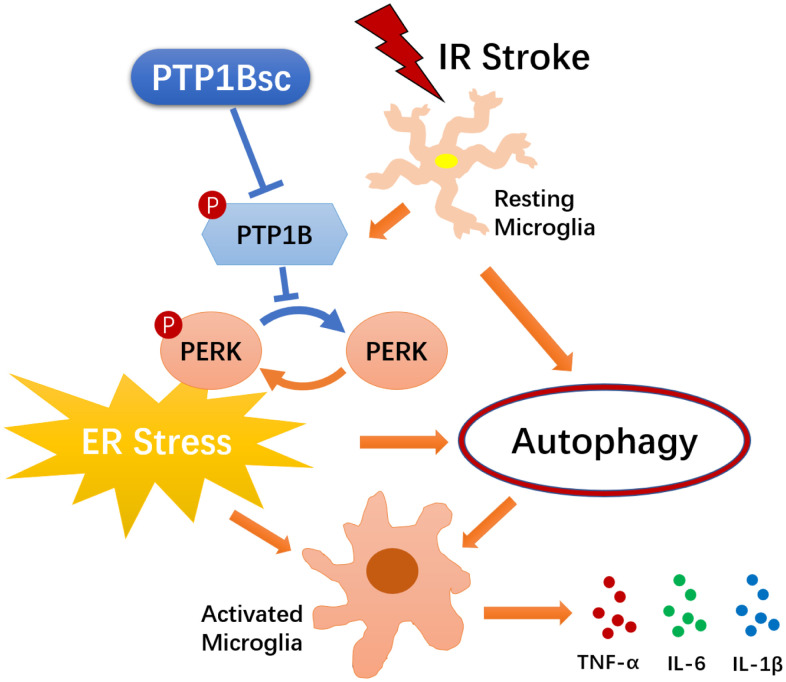
**Schematic diagram of the indicated molecular mechanisms underlying the protective effects of PTP1B inhibitor treatment against cerebral ischemia/reperfusion injury.** PTP1B inhibitor treatment alleviated cerebral IR-induced microglial ER stress as well as downstream autophagy, and ultimately mitigated deleterious activation of microglia. PTP1B inhibitor attenuated microglial ER stress possibly by inhibiting PERK signaling. PTP1Bsc = PTP1B inhibitor sc-222227.

In summary, as illustrated in Figure 8, our study suggests that the PTP1B inhibitor, sc-222227, is able to reduce cerebral IR injury-induced deleterious microglial activation and subsequent neuroinflammation by modulating the ER stress-autophagy axis via PERK signaling in microglia. Intracerebroventricular administration of PTP1B inhibitor effectively protects against cerebral IR injury. These findings provide novel insight into the molecular association between PTP1B, ER stress-autophagy axis, microglial activation, and neuroinflammation, suggesting that PTP1B is an important factor in regulating microglia function. Moreover, our study sheds light on novel therapeutic strategies that target microglial PTP1B for cerebral ischemic stroke treatment.

## MATERIALS AND METHODS

### Reagents

The PTP1B inhibitor, sc-222227, was purchased from Santa Cruz Biotechnology, Inc. (Santa Cruz, CA, USA). The autophagy inhibitor, N(3)-methyladenine (3-MA) and the ER stress inhibitor, 4-phenylbutylamine (4-PBA), were purchased from Sigma (Sigma, St. Louis, MO, USA). The protein kinase R-like endoplasmic reticulum kinase (PERK) activator, CCT020312, was purchased from Merck Millipore (Billerica, MA, USA). The PERK inhibitor, GSK2606414, was purchased from Sigma. Antibodies were obtained from the following sources: CD11b/c, PTP1B, inositol requiring enzyme-1 (IRE1), p-IRE1, activating transcription factor 6 (ATF6), neuronal nuclear antigen (NeuN), ionized calcium binding adapter molecule 1 (Iba-1), heavy chain binding protein (Bip), glyceraldehyde-3-phosphate dehydrogenase (GAPDH), and β-actin were purchased from Abcam (Cambridge, MA, USA); LC3-I/II and beclin-1 were purchased from Cell Signaling (Danvers, MA, USA); glial fibrillary acidic protein (GFAP) was purchased from Santa Cruz Biotechnology, Inc.; primary antibody for p-PERK (Thr982), PERK, p-eIF2α (Ser51), eIF2α, CD16/32, CD206, and secondary antibodies were purchased from Invitrogen (Carlsbad, CA, USA); and donkey anti-rabbit IgG, rabbit anti-goat IgG, and goat anti-mouse IgG were purchased from Invitrogen.

### Animals

Male Wistar rats weighing 200-250 g were obtained from Shanghai SLAC Laboratory Animal Co., Ltd. (Shanghai, China). Animals were housed under conditions of constant temperature and humidity, and kept on a 12 h light, 12 h dark cycle. Food and water were available *ad libitum*. All procedures for handling animals were performed according to the protocols approved by the Institutional Animal Care Committee of the First Affiliated Hospital (Zhejiang University School of Medicine). The total number of animals used in the study and the number of animals included and excluded for each experiment are shown in Supplementary Figure 1.

### MCAO and reperfusion

A transient MCAO model using the intraluminal suture method was established as previously described [56, 57]. Briefly, rats were anesthetized with intraperitoneal injection of pentobarbital sodium (50 mg/kg). A normothermic range of body temperature (37-38° C) was maintained with a temperature-controlled heating pad. A midline neck incision was made, followed by isolation of the right common, internal, and external carotid arteries. A 4/0 monofilament nylon with a silicone-beaded tip was inserted into the right internal carotid artery through the external carotid artery to block the blood supply of the right middle cerebral artery. Laser Doppler flowmetry (PeriFlux 5000; Stockholm, Sweden) was used to monitor the blood flow of the right middle cerebral artery. Successful ischemia was defined as reduction in blood flow of > 25% from the baseline. After 2 h of occlusion, the monofilament was withdrawn to initiate reperfusion. The sham groups were objected to the same operation without insertion of a monofilament.

### Intracerebroventricular drug administration

The PTP1B inhibitor, sc-222227 (Santa Cruz Biotechnology, Inc.), was dissolved in DMSO and diluted with 0.9 saline to a final concentration of < 0.1% DMSO. Anesthetized rats were placed in a stereotaxic frame, and sc-222227 (5 and 10 μM) was injected into the right cerebral ventricle with a Hamilton syringe using the following coordinates: 3 mm rostral to the bregma; 2 mm lateral to the midline; and 2 mm ventral to the skull surface. The injection rate was 0.2 μL/min, and the needle was left in place for 5 min after injection before being slowly withdrawn. After drug administration, MCAO and reperfusion were performed immediately, as described above. Both sham and IR groups were administered vehicle (DMSO) intracerebroventricularly.

### Neurologic score and motor assessment scale

Neurologic status was assessed 24 h after reperfusion according to a 21-point Garcia test score system, as described previously [24]. Briefly, seven tests were included in the score system: spontaneous activity; axial sensation; vibrissae proprioception; symmetry of limb movement; lateral turning; forelimb outstretching; and climbing. Each test was scored between 0 (worst) and 3 (best) for a total score of 21. Motor function was evaluated according to a 10-point score system (number of successful forepaw placements out of 10 consecutive vibrissae-elicited excitation), as previously reported [36].

### TTC staining and quantification of infarct volume

Rats were euthanized with an overdose of anesthesia 24 h post-reperfusion and quickly decapitated. The brain was immediately removed and weighed, and sectioned coronally at 2-mm intervals. Then, the slices were stained with 2% 2,3,5 TTC for 15 min at 37° C. Digitalized slice images and the infarct areas were analyzed by Image J (version 1.49; NIH, Bethesda, MD, USA). The viable part of the brain slice was red, while the infarct lesion was defined as complete lack of staining with TTC. The infarct volume was calculated by multiplying the added infarct areas of each slice, as follows: (infarct volume/whole brain volume) × 100%.

### Assessment of brain water content

Immediately after removal, the brain weight (wet weight) was assessed. Then, brain slices after TTC staining were dried at 110° C for 48 h and the dry brain weight was assessed. The brain water content was calculated as follows: (wet weight-dry weight)/wet weight × 100%.

### Flow cytometry analysis of the proportion of Iba-1(+)/PTP1B(+), GFAP(+)/PTP1B(+), and NeuN(+)/ PTP1B(+) double-positive cells

Rat brain tissue was cut into 2~3 mm^3^ pieces and rinsed with cold PBS. Then, 5 mL of digestive solution (125 U/mL of collagenase type XI, 50 U/mL of hyaluronidase type 1-S, 100 U/mL of DNase I, and 500 U/mL of collagenase type I) was added into the DMEM medium containing 10% FBS of the same volume, filtered through a 200-mesh sieve, centrifuged at 300 g for 5 min, and fixed at room temperature with 2% paraformaldehyde for 20 min.

Triton X-100 (0.2%) was used to lyse the membrane at room temperature for 15 min, and re-suspended in 500 mL of PBS. Antibodies against NeuN (1:100), Iba-1 (1:100), and GFAP (1:100) were added and incubated at 4° C for 1 h. PTP1B antibody (1:100) was added to each tube and incubated at 4° C for 8 h. Donkey anti-rabbit IgG (for PTP1B, 1:500), rabbit anti-goat IgG (for Iba-1, 1:500), and goat anti-mouse IgG (for GFAP and NeuN, 1:500) were added. Flow cytometry was used to detect PTP1B in microglia cells [PTP1B(+)/ Iba-1(+) double-positive], PTP1B in astrocytes [GFAP(+) / PTP1B(+) double-positive], and PTP1B in nerve cells [NeuN(+)/PTP1B (+) double-positive] in rat brain tissues.

### Rat primary microglial cell isolation

Primary microglia cells were obtained from 1-3-day-old rat pups, as previously reported [58, 59]. Briefly, neonatal rat cerebral cortices were minced into small pieces, digested in DMEM, and centrifuged (300 × *g* for 10 min). The precipitate was resuspended in DMED with 5% fetal calf serum, 10% FBS, and 0.05 mg/ml of gentamycin (Invitrogen) in an incubator at 37° C in 5% CO_2_ and 95% air. The cellular debris, non-adherent cells, and the supernatant were removed after 2 days, and the mixed cells were cultured for 8-10 days. Then, by shaking flasks on an orbital shaker at 65 rpm for 4-6 h at 37° C, microglial suspensions were harvested. The trypan blue test confirmed that the cell viability was > 95%. The purity of the microglia was > 99%, and confirmed by immunostaining for the microglia/macrophage marker, CD11b/c (Abcam).

### Establishment of stable GFP-LC3-expressing BV-2 cells

The establishment of stable GFP-LC3-expressing BV-2 cells was performed, as previously described [60]. Briefly, BV-2 cells were cultured in DMEM containing 10% FBS. Cells were incubated at 37° C in a 5% CO_2_ incubator. pEGFP-LC3 plasmid was purchased from Addgene (Boston, MA, USA). After amplification by PCR with LA tag polymerase (Takara, Shiga, Japan), the GFP-LC3 gene was cloned into the pCR8/GW/TOPO vector (Invitrogen), then inserted into the pLenti6.3/V5-DEST vector (Invitrogen) using LR clonase (Invitrogen). After construction of lentivirus, BV-2 cells were infected by the lentivirus (MOI = 5) together with 8 μg/mL of polybrene and cultured for 72 h. Cell selection were performed with 4 μg/mL of blasticidin (Invitrogen) for several days.

### *In vitro* oxygen glucose deprivation/reoxygenation (OGD/R) and drug treatments

Primary microglial cells and stable GFP-LC3-expressing BV-2 cells were maintained in glucose-free medium in an anaerobic chamber (Thermo Fisher Scientific, Waltham, MA, USA) filled with 94% N_2_, 1% O_2_, and 5% CO_2_ at 37° C for 3 h. Then, cells were returned to normal cell culture incubators (95% air and 5% CO_2_) with normal medium. After 24 h of re-oxygenation, the OGD/R-treated conditioned medium was collected, and the supernatant without OGD/R treatment served as the sham control. The PTP1B inhibitor, sc-22227 (1 and 2 μM; Santa Cruz Biotechnology, Inc.), the PERK activator, CCT020312 (200 nM; Millipore), the PERK inhibitor, GSK2606414 (100 nM; Sigma), 4-PBA (4 mM; Sigma), and 3-MA (1 mM; Sigma) were added to microglial cultures 2 h before OGD/R treatment.

### GFP-LC3 puncta formation assay

GFP-LC3 puncta in stable GFP-LC3-expressing BV-2 cells were detected using a laser confocal microscope (A1l Nikon, Tokyo, Japan). The number of cells with GFP-LC3 puncta among all GFP-LC3-expressing BV-2 cells were counted. A minimum of 100 cells from six randomly selected fields were analyzed by Image J (version 1.49).

### Western blot

Western blots were performed to detect protein levels in the ipsilateral cerebral cortex and primary microglia cells. The ipsilateral cerebral cortex tissues were homogenized and lysed with RIPA buffer (Thermo Fisher Scientific) with protease and phosphatase inhibitor cocktails (Abcam) and treated cells were lysed using a Mammalian Cell Lysis kit (Sigma). The extracted proteins were then separated by 10% sodium dodecyl sulfate (SDS)-polyacrylamide and electrically transferred to PVDF membranes (Millipore). The membranes were then blocked with TBST with 5% non-fat dry milk for 1 h at room temperature. The western blots were probed with primary antibodies recognizing the following proteins and further incubated with corresponding secondary antibody (1:10000; Invitrogen): PTP1B (1:1000; Abcam); p-PERK (Thr982, 1:1000; Invitrogen); PERK (1:1000; Invitrogen); p-eIF2α (Ser51, 1:1000; Invitrogen); eIF2α (1:1000; Invitrogen); p-IRE1 (Ser724, 1:1000; Abcam); IRE1 (1:2000; Abcam); ATF6 (1:2000; Abcam); LC3-I/II (1:1000; Cell Signaling); beclin-1 (1:1000; Cell Signaling); β-actin (1:5000; Abcam); and GAPDH (1:5000; Abcam). The levels of protein expression were analyzed using Image J software (version 1.49) normalized to β-actin and GAPDH. Phosphorylated protein levels were evaluated compared to total protein levels.

### Immunohistochemistry, double immunofluorescence, and TUNEL staining

For immunohistochemistry, immunofluorescence, and the TUNEL assay, rats were anesthetized and perfused through the ascending aorta with 0.9% saline followed by 4% paraformaldehyde after cerebral IR injury. After decapitation, brains were dehydrated with sucrose prior to embedding with paraffin.

Immunohistochemistry was performed in the ipsilateral hemisphere in paraffin-embedded coronal sections (6 μm), as reported previously [61]. Briefly, after de-waxing, the sections were washed with PBS (pH 7.4), processed with 3% hydrogen peroxide, and washed with PBS. Sections were then incubated with primary antibody to detect CD11b/c (1:1000; Abcam) overnight at 4° C. The sections were then retrieved and washed with PBS, followed by addition of secondary antibody (1:5000; ZSGB-BIO Company, Beijing, China) for incubation at room temperature for 30 min. Sections were then washed with PBS, and diaminobenzidine (DAB) chromogenic reagent (ZSGB-BIO Company) was used for developing sections. The reaction was terminated by tap water and hematoxylin was used for counterstaining the nucleus, followed by washing and bluing. Finally, sections were dehydrated and sealed with neutral gum, visualized, and photographed using a microscope (Leica, Heerbrugg, Germany).

For the double immunofluorescence assay, sections were first blocked with 10% donkey serum for 2 h to avoid non-specific staining. Then, sections were incubated with primary antibody against PTP1B (1:50; Abcam), NeuN (1:300; Abcam), Iba-1 (1:500; Abcam), GFAP (1:1000; Santa Cruz Biotechnology, Inc.), p-PERK (Thr982, 1:200; Invitrogen), CD16/32 (1:100; Invitrogen), CD206 (1:100; Invitrogen), p-IRE1 (1:100; Abcam), and Bip (1:100; Abcam) at 4° C for overnight. Then, sections were incubated with secondary antibodies (Invitrogen) at room temperature for 2 h after washing in PBS. Samples were counterstained with 4,6-diamidino-2-phenylindole dihydrochloride (DAPI) for 5 min, then observed and photographed with a laser confocal microscope (A1; Nikon).

To detect apoptotic neurons cells, immunofluorescence and TUNEL staining were performed. Briefly, sectioned slides were washed in TBS (50 mM Tris-HCl [pH 7.4] and10 mM NaCl) plus 0.3% Triton X-100 with gentle agitation and blocked in 10% normal serum in TBS for 2 h at room temperature. Slides were then incubated with anti-NeuN antibody (1:300; Abcam) overnight at 4° C. Then, slides were incubated with secondary antibody for 2 h at room temperature after rinsing with TBS. Sections were then stained using a TUNEL kit (Keygenbiotech, Nanjing, China) according to the manufacturer’s instructions. The sections were incubated with DAPI for 10 min, and finally observed and photographed with a laser confocal microscope (A1; Nikon). Apoptotic neurons were identified by green TUNEL dots located in red neurons with a blue nucleus.

For the immunohistochemistry assay, five views from the penumbra site (-3.0 mm ± 0.5 mm from the bregma) were assessed for each experiment, and for immunofluorescence and TUNEL assays, six views from the same penumbra site as above were assessed for each experiment by a pathologist blinded to the experimental conditions.

### Measurements of mRNA levels

Total RNA was extracted from ipsilateral cerebral cortex tissues and primary microglia cells by TRIzol reagent (Takara, Kyoto, Japan). Samples were then reverse-transcribed to cDNA using a cDNA Synthesis kit (Takara). Then, cDNA was amplified using a SYBR Premix Ex Taq kit (Takara) on a 7500 Real-Time PCR System (Applied Biosystems, Carlsbad, CA, USA). All procedures were performed according to the manufacturers’ instructions. Data were normalized to β-actin and expressed as a fold-change compared to the control. The primers used for the amplification are shown in Table 1 (Sangon Biotech Co., Ltd., Shanghai, China).

**Table 1:** Primers used for qRT-PCR.

**Primers**	**Forward (5’-3’)**	**Reverse (5’-3’)**
**TNF-α**	TGTGGAACTGGCAGAGGA	ACAGAAGAGCGTGGTGGC
**IL-1β**	CAAATCTCACAGCAGCATCTC	AGGACGGGCTCTTCTTCA
**IL-6**	GCCACTGCCTTCCCTACT	CACAACTCTTTTCTCATTTCCA
**CCL2**	TGTTGTTCACAGTTGCTGCCTG	GTGCTGAAGTCCTTAGGGTTGAT
**β-actin**	CAAGTGGGTGGCATAGAGG	ATGACGAAGAGCACAGATGG

### Enzyme-linked immunosorbent assay (ELISA)

The levels of secreted pro-inflammatory cytokines, including tumor necrosis factor-alpha (TNF-α) and chemokine (C-C motif) ligand 2 (CCL2) in supernatants collected from rat primary microglia were detected by commercially available ELISA kits (eBioScience, San Diego, CA, USA), according to the manufacturer’s instructions. The data represent results obtained from four independent tests.

### Proximity ligation assay (PLA) and quantification of PLA signals

PLAs were performed to detect protein interactions, as previously described [34, 62, 63]. Briefly, after PTP1B inhibitor treatment (sc-22227, 2 μM; Santa Cruz Biotechnology, Inc.) and the OGD/R insult, primary microglia were incubated with primary antibodies against PTP1B (1:100; Abcam) and p-PERK (Thr982, 1:200; Invitrogen), then with a pair of PLA probes (Sigma). Probe ligation (Sigma), signal amplification (Sigma), and mounting with DAPI (Sigma) were performed according to the manufacturer’s instructions. Representative images were obtained with a Z1 inverted microscope (Carl Zeiss, Göttingen, Germany). Images were analyzed using ImageJ software (version 1.49). Quantification of the PLA signal was expressed as the average number of PLA puncta/blobs per DAPI-positive nuclei in the sample field.

### Statistical analysis

All data were analyzed using SPSS (version 19.0; SPSS, Inc., Chicago, IL, USA). Values are expressed as the mean ± SEM. The non-paired t test was used to determine the significance of differences between two groups. P < 0.05 was considered to be statistically significant.

## Supplementary Material

Supplementary Figure 1
