# Supplementary material for: PTP1B inhibitor alleviates deleterious microglial activation and neuronal injury after ischemic stroke by modulating the ER stress-autophagy axis via PERK signaling in microglia
Source: Aging (Albany NY). 2021 Jan 20;13(3):3405–27. doi: 10.18632/aging.202272 (PMC7906217; doi:10.18632/aging.202272)
Supplement: Supplementary Figure 1 [file aging-13-202272-s001.pdf]

SUPPLEMENTARY FIGURE

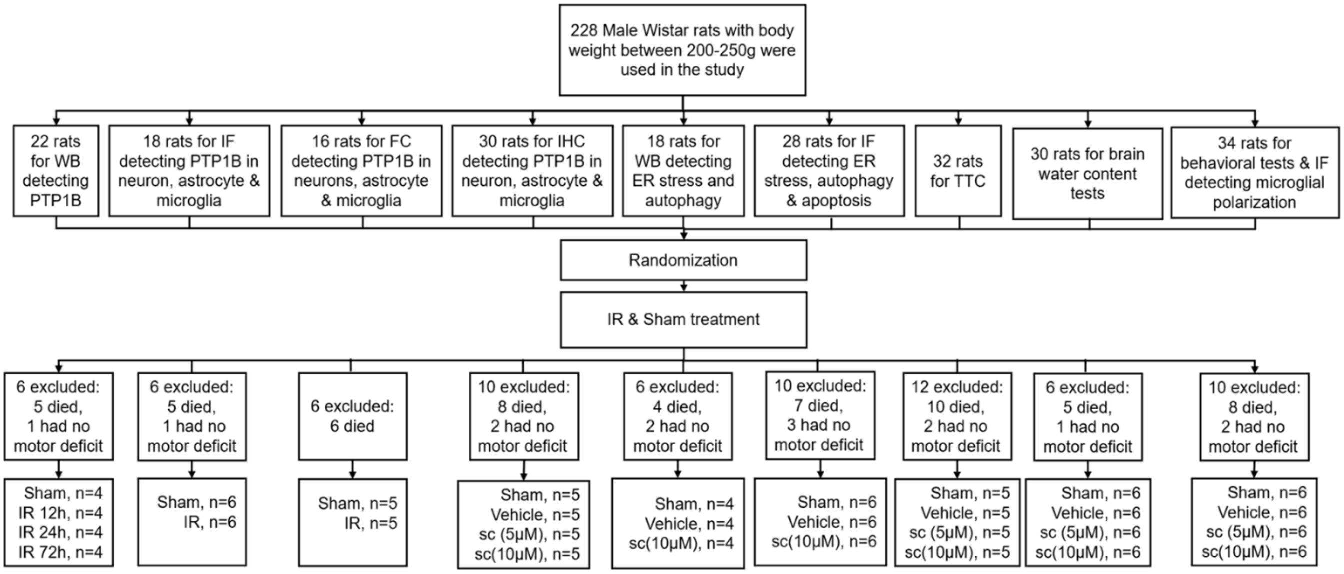

Supplementary Figure 1. Total number of animals used in the study and the number of animals included and excluded in each experiment.
